# Aortic Valve Calcium Associates with All-Cause Mortality Independent of Coronary Artery Calcium and Inflammation in Patients with End-Stage Renal Disease

**DOI:** 10.3390/jcm9020607

**Published:** 2020-02-24

**Authors:** Lu Dai, Oscar Plunde, Abdul Rashid Qureshi, Bengt Lindholm, Torkel B. Brismar, Leon J. Schurgers, Magnus Söderberg, Jonaz Ripsweden, Magnus Bäck, Peter Stenvinkel

**Affiliations:** 1 Division of Renal Medicine and Baxter Novum, Department of Clinical Science, Intervention and Technology, Karolinska Institutet, 141 86 Huddinge, Stockholm, Sweden; lu.dai@ki.se (L.D.); tony.qureshi@ki.se (A.R.Q.); bengt.lindholm@ki.se (B.L.); 2 Department of Medicine, Karolinska Institutet, 171 76 Solna, Stockholm, Sweden; oscar.persson@ki.se (O.P.); Magnus.Back@ki.se (M.B.); 3 Division of Medical Imaging and Technology, Department of Clinical Science, Intervention and Technology, Karolinska Institutet, 141 86 Huddinge, Stockholm, Sweden; torkel.brismar@gmail.com (T.B.B.); jonaz.ripsweden@ki.se (J.R.); 4 Department of Radiology, Karolinska University Hospital, 141 86 Huddinge, Stockholm, Sweden; 5 Department of Biochemistry, Cardiovascular Research School Maastricht, Maastricht University, 6229 ER Maastricht, The Netherlands; l.schurgers@maastrichtuniversity.nl; 6 Cardiovascular, Renal and Metabolism Safety, Clinical Pharmacology and Safety Sciences, AstraZeneca R&D, 431 83 Molndal, Gothenburg, Sweden; Magnus.Soderberg@astrazeneca.com; 7 Theme Heart and Vessels, Division of Valvular and Coronary Heart Diseases, Karolinska University Hospital, 141 86 Huddinge, Stockholm, Sweden

**Keywords:** aortic valve calcium, end-stage renal disease, coronary artery calcium, calcification, mortality

## Abstract

Background: Aortic valve calcium (AVC) and coronary artery calcium (CAC) are common complications in end-stage renal disease (ESRD). We investigated the prognostic significance of overlapping presence of AVC and CAC, and whether AVC was associated with all-cause mortality independent of the presence of CAC in ESRD. Methods: 259 ESRD patients (median age 55 years, 67% males) undergoing cardiac computed tomography were included. Framingham risk score (FRS), presence of cardiovascular disease (CVD), statin use, nutritional status and other relevant laboratory data were determined at baseline. During follow-up for median 36 months, 44 patients died, and 68 patients underwent renal transplantation. Results: The baseline overlap presence of AVC and CAC was 37%. Multivariate regression analysis showed that FRS (odds ratio (OR) 2.25; 95% confidence interval (95% CI), 1.43–3.55) and CAC score (OR (95% CI), 2.18 (1.34–3.59)) were independent determinants of AVC. In competing-risk regression models adjusted for presence of CAC, inflammation, nutritional status, CVD, FRS and statin use, AVC remained independently associated with all-cause mortality (sub-hazard ratio (95% CI), 2.57 (1.20–5.51)). Conclusions: The overlap of AVC and CAC was 37% in this ESRD cohort. AVC was associated with increased all-cause mortality independent of presence of CAC, traditional risk factors and inflammation.

## 1. Introduction

Patients with chronic kidney disease (CKD) are at high risk of cardiovascular morbidity and mortality [1,2]. Aortic valve calcium (AVC) has been reported to occur 10–20 years earlier in CKD patients with a higher prevalence (28–55%) than that reported in the general population [3,4,5,6,7,8]. The association of cardiac valve (mitral and aortic) calcification and clinical outcomes has been studied in patients with end-stage renal disease (ESRD). However, results are rather discrepant, largely due to the heterogeneity of adjusted confounders [3,9,10]. Despite the fact that premature AVC is believed to share a similar risk profile as the more frequently detected coronary atherosclerosis [11], the prognostic implication of overlapping prevalence of AVC and coronary artery calcium (CAC) has not been investigated in ESRD. Moreover, low grade chronic inflammation is highly prevalent in CKD, and in accordance with the inflammation-catalyst hypothesis [12], inflammation plus cardiac valve calcification would increase the risk of mortality in haemodialysis (HD) patients [9].

Cardiovascular risk stratification is crucial for illustrating factors associated with survival in patients with CKD. To better understand the prognostic significance of AVC (evaluated by computed tomography (CT) scan) and clinical outcome, we analysed the data from an observational ESRD cohort with five-year follow up. In addition, since inflammation is involved in the aetiology of both AVC and CAC, we assessed the association of the combined presence of AVC, CAC and inflammation with all-cause mortality in ESRD. Finally, in a subset of 102 patients, we related AVC to the extent of media calcification scoring in uremic arterial biopsies.

## 2. Methods and Patients

### 2.1. Patient Selection

We analysed 259 ESRD patients, who underwent cardiac CT scan of AVC and CAC score at Karolinska University Hospital, Huddinge, including 139 CKD5 non-dialysis (CKD5-ND) and 120 dialyzed (CKD5-D) patients receiving peritoneal dialysis (PD; *n* = 85) or HD (*n* = 35) (Supplementary Materials Table S1 and Figure S1). The patients (median age 55 years, 67% male, 18% diabetes and 20% cardiovascular disease (CVD)) were recruited from ongoing cohorts as described below. Exclusion criteria were age < 18 years, acute renal failure, signs of overt infection and unwillingness to participate. No patients with stent or valve implantation were recruited in this cohort study. None of the patients were lost to follow up. Informed consent was obtained from each patient, and the study protocols were approved by the Ethics Committee of the Karolinska Institute at the Karolinska University Hospital Huddinge, Stockholm, Sweden and conducted in adherence to the Declaration of Helsinki.

CKD5-D patients (*n* = 120) were enrolled from two cohort studies with follow-up in PD and HD, respectively [13]. PD patients (median dialysis vintage 11.4 months) received either biocompatible glucose-based or amino acid-based or, for the long dwell, icodextrin-based solutions. Causes of ESRD included chronic glomerulonephritis (*n* = 16), hypertension and renovascular disease (*n* = 5), diabetic nephropathy (*n* = 9), autosomal dominant polycystic kidney disease (ADPKD) (*n* = 6) and other or unknown causes (*n* = 48). HD patients (median dialysis vintage 12.7 months) were treated by conventional maintenance HD or hemodiafiltration. Causes of ESRD included chronic glomerulonephritis (*n* = 15), hypertension and renovascular disease (*n* = 4), ADPKD (*n* = 4) and other or unknown causes (*n* = 13).

CKD5-ND patients (*n* = 139) were enrolled from a cross-sectional cohort with follow-up with CKD5 patients prior to initiation of dialysis [14] and from an ongoing cohort study in CKD5 patients listed as living donor kidney transplantation (LD-Rtx) recipients [15]. The aetiologies of ESRD included chronic glomerulonephritis (*n* = 46), hypertension and renovascular disease (*n* = 16), diabetic nephropathy (*n* = 20), ADPKD (*n* = 23) and other or unknown causes (*n* = 34). The median estimated glomerular filtration rate (CKD-EPI) was 6 (5–8) mL/min/1.73 m^2^.

### 2.2. Aortic Valve Calcium and Coronary Artery Calcium by Computed Tomography (CT) Imaging

All patients underwent non-contrast multi-detector cardiac CT (LightSpeed VCT or Revolution CT; GE Healthcare, Milwaukee, WI, USA) scanning with standard ECG-gated protocol, to evaluate AVC and CAC Agatston scores. AVC-scores were computed using the Agatston CAC-scoring method from non-contrast cardiac CT scans. AVC was determined as the sum of total calcifications in the aortic valve area including calcifications within the valve leaflets as well in the aortic wall immediately connected to the leaflets. The details of CAC-scoring was described previously [16]. Presence of AVC and CAC was defined as total AVC score >0 and CAC score >0, respectively.

### 2.3. Histological Assessment of Arterial Media Calcification

The extent and severity of media calcification was evaluated by a pathologist in uremic vascular biopsies obtained from inferior epigastric arteries in 102 LD-Rtx recipients [15]; details are presented in Supplementary Materials Text S1.

### 2.4. Biochemical Assessments

Blood laboratory biochemical measurements including high-sensitivity C-reactive protein (hsCRP) and other markers of interest were analysed by routine methods or commercial kits; details are presented in Supplementary Materials Text S1.

### 2.5. Clinical Data Collection

Cardiovascular disease (CVD) was defined based upon clinical history or signs of ischemic cardiac disease and/or presence of cerebrovascular disease and/or peripheral vascular disease. Nutritional status was evaluated by subjective global assessment (SGA) score (well-nourished (SGA score 1), mild-malnourished (SGA score 2), moderate-malnourished (SGA score 3) and severe-malnourished (SGA score 4)) [17]. For simplicity, patients were combined in two groups as malnourished group (SGA score > 1) and well-nourished group (SGA score = 1). Handgrip strength (HGS) was measured in the non-fistula arm using the Harpenden dynamometer (Yamar, Jackson, MI, USA) and repeated three times, and the largest value was recorded and expressed in kg; HGS was expressed in % of healthy individuals with same sex in analyses. Body mass index (BMI) was calculated as weight in kilograms divided by the square of height in meters. The details of augmentation index (AIx%), an assessment of arterial stiffness and skin autofluorescence (SAF) (a marker of advanced glycation end-products (AGEs)) are described in Supplementary Materials Text S1.

### 2.6. Framingham Risk Score (FRS)

Framingham risk score (FRS), an estimation of 10-year risk of developing CVD, was calculated from sex- and age-stratified formulas with scores including systolic blood pressure (SBP), diabetes, anti-hypertensive medication, total cholesterol, high-density lipoprotein (HDL) cholesterol and smoking [18].

### 2.7. Statistical Analyses

Data were presented as median (interquartile range, IQR) or percentage, as appropriate. Statistical significance was set at the level of *p* <0.05. Comparisons between more than three groups were performed with the Kruskal–Wallis test for continuous variables followed by Dunn´s test and with Chi-square test for nominal variables. Non-parametric univariate Spearman rank correlation analysis was applied to determine correlations between two variables. Multivariate logistic regression analysis was applied to examine parameters associated with presence of AVC. Patients were followed from the inclusion date until renal transplantation or death or completing 60-month follow-up. Causes of death were established with the death certificate issued by the attending physician. We used Fine and Gray models for competing-risk regression models with renal transplantation as a competing risk to establish cumulative incidence curves [19]. Inflammation was defined as hsCRP >10 mg/L. Risk estimates for patients with AVC score >0 were presented as sub-hazard ratios (sHR), with patients with AVC score = 0 as reference. Statistical analyses were performed using statistical software SAS version 9.4 (SAS Campus Drive, Cary, NC, USA) and Stata 15.1 (Stata Corporation, College Station, TX, USA).

## 3. Results

### 3.1. Baseline Characteristics

Characteristics of the patients according to the presence of AVC are shown in Table 1. Among 259 patients, AVC was present in 100 (39%) patients, with a median AVC score of 90 (21–242) Agatston units (AU) and median CAC score of 875 (328–2058) AU. Patients with AVC were older and had more comorbidities. They were more often smokers and users of beta-blockers and statins. They had higher BMI, FRS, triglycerides, hsCRP, interleukin-6 (IL-6), CAC score, AGEs and AIx% and lower %HGS and serum albumin.

**Figure 1:**
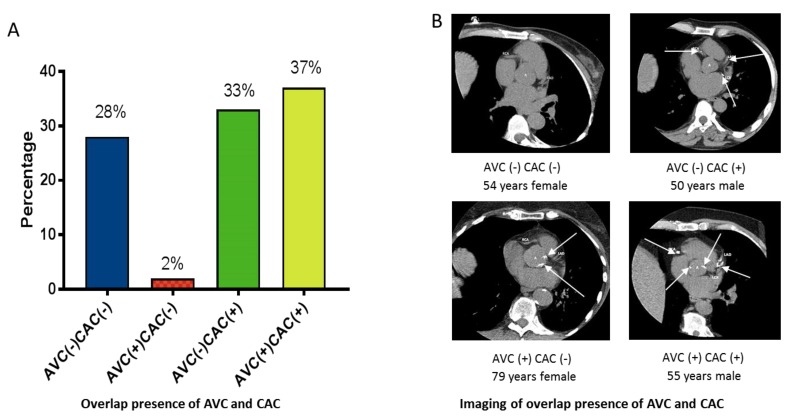
Presence of aortic valve calcium (AVC) and coronary artery calcium (CAC) among 259 ESRD patients. (**A**) Prevalence of four groups of patients according to presence (+) or not (-) of AVC or CAC. (**B**) Computed tomography imaging representing the four groups of patients.

To investigate the overlap prevalence of AVC and CAC, patients were further classified into four groups based on presence (+) or absence (-) of AVC and CAC at baseline (Figure 1): AVC (−) CAC (−), *n* = 72, 28%; AVC (+) CAC (−), *n* = 5, 2%; AVC (−) CAC (+), *n* = 87, 33%; AVC (+) CAC (+), *n* = 95, 37% (Figure 1A); representative CT scan imaging from the four groups of patients are shown in Figure 1B. Patients with an overlap of AVC (+) and CVC (+) were older and had more comorbidities. They were more often smokers and users of beta-blockers and statins. They had higher BMI, FRS, IL-6, AGEs and AIx%, lower %HGS, and higher HDL and hsCRP than patients with AVC (+) CAC (−) (Supplementary Materials Table S2).

### 3.2. Stratified Distribution of Calcification in Different Sites

**Figure 2:**
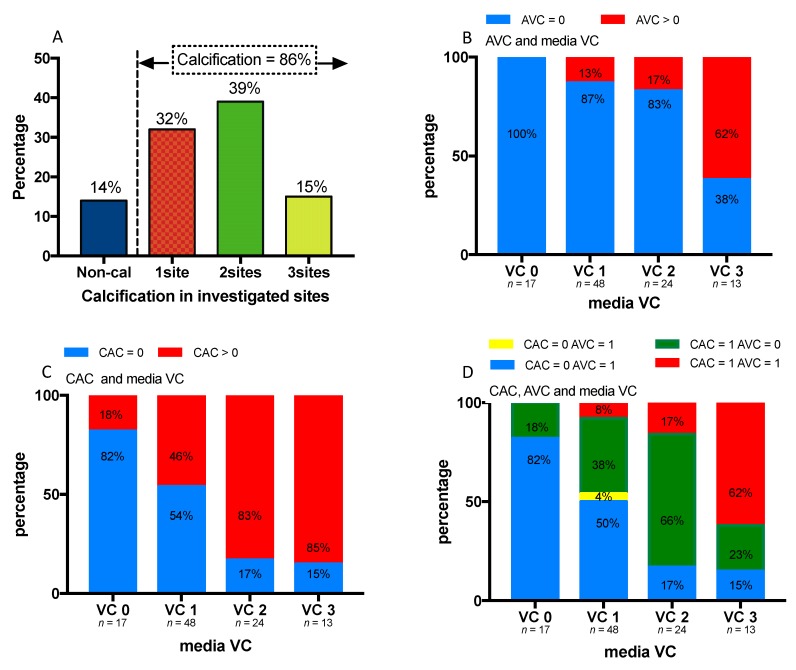
Prevalence of calcification at three sites, inferior epigastric artery (media vascular calcification, VC), aortic valve (AVC) and coronary artery (CAC) among 102 ESRD patients who underwent both arterial biopsies and cardiac CT imaging. (**A**) Prevalence of calcification at 0, 1, 2 or 3 of the three sites. (**B**) Prevalence of AVC with severity of media VC. (**C**) Prevalence of CAC with severity of media VC. (**D**) Prevalence of combined presence of CAC and AVC with severity media VC.

In a sub-analysis of 102 patients available with scoring of medial calcification in the epigastric artery, AVC and CAC, we checked the concurrent calcification in these three investigated sites (Figure 2). Eighty-six percent of the patients had signs of calcification, among which 32% had calcification in one site, 39% in two sites and 15% in all three sites (Figure 2A). The prevalence of AVC, CAC, and the overlap of AVC and CAC increased along with increased severity of medial calcification (Figure 2B–D).

### 3.3. Univariate Correlations Between Presence of AVC and Other Variables

The presence of AVC showed a positive correlation with age (rho = 0.52), presence of CVD (rho = 0.19), diabetes (rho = 0.24), smoking (rho = 0.13), beta-blocker (rho = 0.23) and statin (rho = 0.20) use, FRS (rho = 0.52), hsCRP (rho = 0.29), IL-6 (rho = 0.43), triglyceride (rho = 0.15), AGEs (rho = 0.25), AIx% (rho = 0.23) and CAC score (rho = 0.59), and a negative association with %HGS (rho = −0.34) and serum albumin (rho = −0.28) (Supplementary Materials Table S3).

### 3.4. Multivariate Analysis of Determinants of AVC

In multivariate logistic regression analysis (Supplementary Materials Table S4), per 1-SD increase of FRS (odds ratio (OR 2.25; 95% confidence interval (95% CI), 1.43–3.55) and per 1-SD increase of CAC score (OR 2.18; 95% CI (1.34–3.59)) were identified as independent determinants of AVC (pseudo-R = 0.29).

### 3.5. Association of AVC and CAC with All-Cause Mortality

During follow-up for median 36 months, 44 (17%) out of 259 patients died and 68 (26%) patients underwent LD-Rtx. Associations of AVC and CAC with all-cause mortality were investigated separately, followed by stratification of AVC according to the presence of CAC. In the crude model, the presence of AVC (AVC = 0 as reference) was significantly associated with all-cause mortality (sHR = 5.82; 95% CI (2.87–11.83), Supplementary Materials Figure S2A). The multivariate competing risk models were adjusted with 1-SD FRS, CVD, inflammation, use of statins and nutritional status (Supplementary Materials Figure S2B–D). In separate modelling, the presence of AVC was significantly associated with all-cause mortality with sHR = 2.86; 95% CI (1.27–6.40) (Supplementary Materials Figure S2B), whereas the presence of CAC (non-calcification CAC = 0 as reference) was not significantly associated with all-cause mortality; sHR = 3.13; 95% CI (0.62–15.76) (Supplementary Materials Figure S2C). Compared with the AVC (−) CAC (−) group as reference, AVC (−) CAC (+) and AVC (+) CAC (+) groups were not significantly associated with all-cause mortality (sHR = 1.56, 95% CI (0.29–8.41) and sHR = 4.09, 95% CI (0.78–22.38), respectively) (Supplementary Materials Figure S2D).

### 3.6. Association of AVC and Inflammation with All-Cause Mortality

Associations of AVC and inflammation with all-cause mortality were first investigated separately, followed by stratification of AVC according to the presence of inflammation. The multivariate competing risk models were adjusted with CAC > 0, per 1-SD increase of FRS, presence of CVD, use of statin and nutritional status (Supplementary Materials Figure S3). In separate modelling, the presence of AVC (AVC (−) as reference) was significantly associated with all-cause mortality with sHR = 2.76; 95% CI (1.31–5.80) (Supplementary Materials Figure S3A), whereas the presence of inflammation (inflammation (-) as reference) was not significantly associated with all-cause mortality, sHR = 1.77; 95% CI (0.87–3.60) (Supplementary Materials Figure S3B). Compared with AVC (−) and inflammation (-) group as reference, the stratified groups of patients with presence of AVC had significantly higher risk of mortality: AVC (+) inflammation (-) with sHR = 6.45, 95% CI (1.19–34.8) and AVC (+) inflammation (+) with sHR = 7.31, 95% CI (1.51–35.41), respectively; patients who were inflamed with no presence of AVC showed no increased mortality with sHR = 3.90, 95% CI (0.74–20.49) (Supplementary Materials Figure S3C).

### 3.7. Association of AVC, CAC and Inflammation with All-Cause Mortality

With AVC = 0 as reference, a multivariate competing risk model, adjusted with CAC >0, inflammation, per 1-SD increase of FRS, presence of CVD, use of statins and nutritional status, was constructed to investigate the association of AVC with mortality (Table 2). In this model, while the presence of AVC was significantly associated with increased mortality risk independent of all the adjusted confounders (sHR = 2.57, 95% CI (1.20–5.51)), neither presence of CAC nor inflammation was associated with increased mortality (sHR = 2.25, 95% CI (0.46–11.01) and sHR = 1.56, 95% CI (0.78–3.13), respectively). In addition, per 1-SD increase of FRS and malnutrition was also associated with increased mortality risk (sHR = 1.64, 95% CI (1.27–2.10) and sHR = 2.15, 95% CI (1.18–3.91), respectively).

## 4. Discussion

Aortic valve calcification, often referred to as aortic valve sclerosis, has an estimated prevalence of 25% in individuals over 65 years of age [6] and may progress into significant aortic valve stenosis causing left ventricular obstruction. The CHS (Cardiovascular Health Study), performed in the general population (adults >65 years), showed that aortic valve sclerosis was associated with 50% higher risk of cardiovascular mortality and 42% increased risk of myocardial infarction [20]. The prospective analysis of the Multi-Ethnic Study of Atherosclerosis (MESA) study in the general population further found that the overlap prevalence of AVC and CAC was 11%. Moreover, controlling for patients with presence of subclinical atherosclerosis (estimated by CAC score) and systemic inflammation, the presence of AVC on CT was associated with 50% higher risk of cardiovascular events and 72% increased risk of coronary events [7]. The association of overlap of AVC and CAC with risk of mortality has not been investigated in ESRD.

Based on previous findings in the general population, our study expands the concurrent limited data on the prognostic importance of AVC in ESRD, taking account into the combined effect of coronary arteriosclerosis and inflammatory status. Our data show that AVC was present in 39% of the investigated ESRD patients and overlapping prevalence of AVC and CAC was 37%; i.e., >3 times higher than the prevalence (11%) reported in the general population [7]. More importantly, the prevalence of AVC was an independent predictor of all-cause mortality after adjustments for the presence of CAC and inflammation, as well as for traditional risk factors represented by FRS. These results point to AVC as a strong risk factor of all-cause mortality independent of concomitant coronary calcification and inflammation in ESRD.

Premature vascular ageing is highly prevalent in CKD [21,22,23]. Cardiac valve calcification is reported to be 4–5 times more common in dialysis patients compared to the general population [5,24,25,26]. Similar to the actively-regulated processes of atherosclerosis and vascular calcification processes, pathogenic features involved in valvular calcification include traditional risk factors, inflammation and disordered bio-mineralization [27]. Although valvular calcification is associated with carotid and coronary atherosclerosis both in dialysis and non-dialysis CKD patients, valvular calcification and systemic atherosclerosis do not always co-exist [3,4,28,29]. Our study show that AVC in ESRD was associated with higher CAC score and aortic arterial stiffness, and that FRS and CAC score were independent determinants of AVC in the multivariate logistic model.

To determine the prevalence and distribution of calcification at different arterial sites, we stratified the cohort, according to the presence of AVC and CAC, with the extent of medial VC in a subgroup of 102 patients with arterial biopsies. The high prevalence (86%) of calcification in different sites confirms the extreme premature vascular ageing process in ESRD. Whereas there was an overall similar trend of progressive calcification of AVC and CAC in accordance with the severity of medial vascular calcification, the proportions of AVC and CAC in the different media vascular calcification groups differed markedly. This suggest a different underlying mechanism of calcification in different vascular beds beyond the common risk profile.

Whereas in models with stratification of AVC and CAC, the combined presence of AVC and CAC did not associate with survival difference as compared to other groups, presence of AVC, but not CAC in individual models, was significantly associated with increased all-cause mortality. The association of AVC with all-cause mortality was found to be independent of CAC and following adjustments also for several other established risk factors that are known to be strongly associated with survival, such as FRS, presence of CVD, inflammation, statin use and nutritional status. Our findings implicate that AVC occur due to a distinct pathological pathway that differ from those involved in CAC.

Since uremic inflammation is associated with atherosclerosis progression and poor prognosis [12,30,31], the combined effect of presence of AVC and inflammation was tested. Our data showed that inflammation did not modify the magnitude of the effect of presence of AVC on mortality risk. In fact, the risk associated with AVC was independent of inflammation, concurring the presence of AVC as a strong risk factor of clinical outcome in ESRD.

Based upon the above-mentioned results, we constructed a regression model to determine the combined effects of AVC, CAC and inflammation on the associations with all-cause mortality. After adjustments for FRS, presence of CVD, statins and nutritional status, the presence of AVC remained strongly associated with all-cause mortality (sHR = 2.57, 95% CI (1.20–5.51)). Conversely, the association of both CAC and inflammation with all-cause mortality lost significance after adjustments (Table 2). Therefore, we speculate that the strong association between AVC and all-cause mortality is beyond the underlying atherosclerosis/arteriosclerosis estimated by CAC and the uremic inflammatory burden.

Several limitations should be taken into account when these results are interpreted. Firstly, our results cannot prove causality due to the observational study design, and since some patients may have developed AVC during the follow-up period, the true association between AVC and mortality might be underestimated when analysing AVC at baseline. Secondly, the sample size is relatively small and in survival analysis, we combined the small subgroup of patients with AVC (+) CAC (−) with the group of patients with AVC (+) CAC (+) in order to reduce the skewed statistic power. Our data, therefore, need confirmation in larger CKD5 cohorts. Thirdly, the current study subjects were comprised with CKD5 dialysis and non-dialysis patients; further studies are thus warranted with homogeneity of the study population. On the other hand, strengths of this study include adjustments for clinically relevant confounders (e.g., traditional risk factors represented by FRS, lipid-lowering medication that could improve the prognosis of AVC and nutritional status and inflammation that are factors influencing survival in CKD5 patients); the long observational period without any patients being lost to follow-up; the robust evaluation of AVC by CT; and a comparable assessment of vascular calcification at cardiac and non-cardiac site in arterial biopsies in a subgroup analysis. 

In summary, we report that 37% of CKD5 patients present both CAC and AVC and that the presence of AVC is associated with all-cause mortality independent of CAC, traditional risk factors, inflammation and nutritional status. Although the mechanisms underlying the observed associations remain to be delineated, the strong predictive value of AVC for mortality suggests that AVC should be included in the standard risk evaluation in ESRD.

## Figures and Tables

**Table 1:** Baseline clinical and biochemical characteristics in 259 ESRD patients according to the presence of AVC score.

	AVC = 0	AVC > 0	*p*-Value
	(*n* = 159, 61%)	(*n* = 100, 39%)	
Demography and clinical characteristics			
Age, years	47 (32–58)	64 (56–72)	<0.001
Male sex, *n* (%)	101 (64)	73 (73)	0.11
Diabetes, *n* (%)	17 (11)	30 (30)	<0.001
CVD, *n* (%)	23 (15)	30 (30)	0.003
Smoker, *n* (%)	9 (6)	13 (13)	0.04
Systolic BP, mmHg	142 (129–153)	145 (132–162)	0.11
Diastolic BP, mmHg	85 (76–92)	80 (75–90)	0.14
FRS, %	7.9 (3.2–17.1)	26.4 (15.0–40.8)	<0.001
Treatment modality			0.04
Non-dialysis	89 (56%)	50 (50%)	
Peritoneal dialysis	44 (28%)	41 (41%)	
Haemodialysis	26 (16%)	9 (9%)	
Nutritional status			
Malnutrition (SGA>1)	53 (33%)	35 (35%)	0.78
BMI, kg/m^2^	24.5 (22.1–26.5)	25.4 (23.4–29.1)	0.008
HGS, % of normal	93 (73–108)	74 (61–86)	<0.001
Biochemical markers			
Haemoglobin, g/L	113 (105–121)	113 (104–121)	0.68
Albumin, g/L	35 (32–38)	32 (28–36)	<0.001
HDL, mmol/L	1.3 (1.1–1.6)	1.2 (1.0–1.6)	0.13
Triglyceride, mmol/L	1.4 (1.0–2.0)	1.6 (1.3–2.2)	0.02
Total cholesterol, mmol/L	4.6 (3.9–5.3)	4.5 (3.6–5.2)	0.46
Calcium, mmol/L	2.3 (2.2–2.4)	2.3 (2.2–2.4)	0.44
Phosphate, mmol/L	1.7 (1.4–2.1)	1.8 (1.5–2.1)	0.31
iPTH, ng/L	255 (170–430)	292 (179–450)	0.42
Inflammatory markers			
hsCRP, mg/L	1.2 (0.5–3.2)	2.9 (1.0–7.8)	<0.001
IL-6, pg/mL	2.0 (0.8–4.9)	5.4 (3.3–9.1)	<0.001
AVC and CAC			
AVC score, AU	0	90 (21–242)	<0.001
CAC score, AU	3 (0–165)	875 (328–2058)	<0.001
Others			
AGEs, AU	3.1 (2.6–3.4)	3.5 (2.9–3.9)	<0.001
AIx, %	20.9 (13.3–28.2)	26.3 (20.0–32.0)	<0.001
Medications			
Ca-Blocker, *n* (%)	76 (48)	55 (55)	0.26
Beta-Blocker, *n* (%)	90 (57)	79 (79)	<0.001
ACEi/ARB, *n* (%)	105 (66)	62 (62)	0.51
Statin, *n* (%)	46 (29)	49 (49)	0.001

Data are presented as median (IQR, interquartile range) for continuous measures and *n* (%) for categorical measures. Abbreviations: ESRD, end-stage renal disease; AVC, aortic valve calcium; CVD, cardiovascular disease; BP, blood pressure; FRS, Framingham CVD risk score; SGA, subjective global assessment; BMI, body mass index; %HGS, hand grip strength, converted to % of sex-matched healthy controls; HDL, high-density lipoprotein; LDL, low-density lipoprotein; iPTH, intact parathyroid hormone; hsCRP, high sensitivity C-reactive protein; IL-6, interleukin-6; AU, Agatston units; CAC, coronary artery calcium; AGEs, advanced glycation end products; AIx (%), augmentation index; ACEi/ARB, angiotensin-converting enzyme inhibitor/angiotensin II receptor blocker.

**Table 2:** Multivariate competing risk regression model of associations among AVC, CAC, inflammation and other factors with 5-year all-cause mortality in 259 ESRD patients.

	sHR (95% CI)	*p*-Value
AVC > 0	2.57 (1.20, 5.51)	0.02
CAC > 0	2.25 (0.46, 11.06)	0.32
1-SD increase of FRS	1.64 (1.27, 2.10)	<0.001
CVD	1.65 (0.90, 3.04)	0.11
Inflammation (hsCRP > 10 mg/L)	1.56 (0.78, 3.13)	0.21
Statin use	1.09 (0.59, 2.02)	0.78
Malnutrition (SGA > 1)	2.14 (1.18, 3.91)	0.01

Abbreviations: AVC, aortic valve calcium; CAC, coronary artery calcium; ESRD, end-stage renal disease; FRS, Framingham CVD risk score; CVD, cardiovascular disease; hsCRP, high sensitivity C-reactive protein; SGA, subjective global assessment.

## Supplementary Materials

Supplementary materials of sub-analyses data are affiliated. The following are available online at https://www.mdpi.com/2077-0383/9/2/607/s1, Figure S1: Flow chart of patient’s recruitment in the cohort study. LD-RTx, living donor kidney transplant; Figure S2: Association of aortic valve calcium (AVC) and coronary artery calcium (CAC) with all-cause mortality in separate and combined models; Figure S3: Association of aortic valve calcium (AVC) and inflammation with all-cause mortality in separate and combined models; Table S1: Baseline clinical and biochemical characteristics in 259 ESRD patients according to modality: non-dialyzed (CKD5-ND) patients, peritoneal dialysis (PD) and hemodialysis (HD); Table S2: Baseline clinical and biochemical characteristics in 259 ESRD patients according to the presence of AVC and CAC; Table S3: Univariate correlations between aortic valve calcium score and other variables (only significant correlations are listed); Table S4: Multivariate logistic regression of factors associated with presence of aortic calcium score.
