# Supplementary material for: Aortic Valve Calcium Associates with All-Cause Mortality Independent of Coronary Artery Calcium and Inflammation in Patients with End-Stage Renal Disease
Source: J Clin Med. 2020 Feb 24;9(2):607. doi: 10.3390/jcm9020607 (PMC7074421; doi:10.3390/jcm9020607)
Supplement: Supplementary file 1 [file jcm-09-00607-s001.pdf]

Supplementary Table 1. Baseline clinical and biochemical characteristics in 259 ESRD patients according to modality: non-dialyzed (CKD5-ND) patients, peritoneal dialysis (PD) and hemodialysis (HD).

	CKD5-ND (N=139, 54%)	HD (N=35, 13%)	PD (N=85, 33%)	p-value
Demography and clinical characteristics				
Age, years	55.0 (43.0-64.5)	46.0 (32.0-61.0)	56.6 (49.0-69.2)	0.002
Male sex, n (%)	91 (65.5%)	27 (77.1%)	56 (65.9%)	0.40
Diabetes, n (%)	25 (18.0%)	2 (5.7%)	20 (23.5%)	0.07
CVD, n (%)	31 (22.3%)	6 (17.1%)	16 (18.8%)	0.72
Smoker, n (%)	10 (7.2%)	2 (5.7%)	10 (11.8%)	0.40
Systolic BP, mmHg	147.0 (135.0-160.0)	140.0 (125.0-160.0)	135.0 (126.0-151.0)	<0.001
Diastolic BP, mmHg	86.0 (79.0-93.0)	84.0 (75.0-93.0)	77.0 (72.0-88.0)	<0.001
FRS, %	14.4 (5.7-26.7)	5.8 (3.6-15.5)	17.8 (5.5-31.3)	0.002
Vascular access, n (%)				
Central venous catheter	-	18 (51%)	-	
Arteriovenous fistula	-	16 (46%)	-	
Arteriovenous graft	-	1 (3%)	-	
Nutritional status				
Malnutrition (SGA>1)	45 (32%)	8 (23%)	35 (41%)	0.13
BMI, kg/m2	25.2 (22.3-28.1)	23.9 (22.3-28.0)	24.7 (22.8-27.1)	0.62
HGS, % of normal	86 (70, 105)	95 (76, 105)	78 (65, 98)	0.02
Biochemical markers				
Hemoglobin, g/L	109.0 (103.0-116.0)	115.0 (110.0-127.0)	117.0 (107.0-125.0)	<0.001
Albumin, g/L	35.0 (31.0-38.0)	36.0 (33.0-39.0)	32.0 (30.0-35.0)	<0.001
HDL, mmol/L	1.2 (1.0-1.5)	1.3 (1.0-1.8)	1.3 (1.0-1.6)	0.42
Triglyceride, mmol/L	1.5 (1.2-2.2)	1.1 (0.8-2.0)	1.5 (1.2-2.2)	0.03
Total cholesterol, mmol/L	4.4 (3.6-5.2)	4.0 (3.5-5.1)	4.8 (4.2-5.6)	0.02
Hemoglobin, g/L	2.3 (2.1-2.4)	2.3 (2.1-2.4)	2.3 (2.2-2.4)	0.54
Albumin, g/L	1.9 (1.6-2.2)	1.7 (1.2-1.8)	1.6 (1.3-1.8)	<0.001
HDL, mmol/L	273.5 (184.0-460.0)	296.0 (173.0-462.1)	256.8 (160.3-412.0)	0.62
Inflammatory markers				
hsCRP, mg/L	1.4 (0.7-4.7)	1.2 (0.4-3.0)	1.7 (0.8-5.6)	0.09
IL-6, pg/mL	3.2 (1.3-7.4)	1.4 (0.4-3.2)	4.5 (2.2-7.9)	0.001
AVC and CAC				

AVC score, AU	0 (0-24)	0 (0-2)	0 (0-137)	0.02
CAC score, AU	82 (0-889)	8 (0-299)	370 (0-1730)	0.01
Others				
AGEs, AU	3.1 (2.6-3.7)	3.1 (2.9-3.4)	3.3 (2.9-3.8)	0.19
Aix, %	23.0 (14.0-30.0)	19.9 (14.2-27.3)	23.0 (17.2-29.4)	0.33
Medications				
Ca-Blocker, n (%)	86 (62%)	13 (37%)	32 (38%)	<0.001
Beta-Blocker, n (%)	91 (66%)	22 (63%)	56 (66%)	0.95
ACEi/ARB, n (%)	106 (76%)	16 (46%)	45 (53%)	<0.001
Statin, n (%)	50 (36%)	9 (26%)	36 (42%)	0.22

Data are presented as median (IQR, interquartile range) for continuous measures, and n (%) for categorical measures; Abbreviations: CVD, cardiovascular disease; BP, blood pressure; FRS, Framingham CVD risk score; SGA, subjective global assessment; BMI, body mass index; %HGS, hand grip strength, converted to % of sex-matched healthy controls; HDL, high-density lipoprotein; LDL, low-density lipoprotein; iPTH, intact parathyroid hormone; hsCRP, high sensitivity C-reactive protein; IL-6, interleukin-6; AU, Agatston units; AVC, aortic valve calcium; CAC, coronary artery calcium; AU, Agatston units; AGEs, advanced glycation end products; AIx(%), augmentation index; ACEi/ARB, angiotensin-converting enzyme inhibitor/ angiotensin II receptor blockers

Supplementary Table 2. Baseline clinical and biochemical characteristics in 259 ESRD patients according to the presence of AVC and CAC

	AVC (-) CAC (-)<br>N=72, 28%	AVC (+) CAC (-)<br>N=5, 2%	AVC (-) CAC (+)<br>N=87, 33%	AVC (+) CAC (+)<br>N=95, 37%	p-value
Demography and clinical characteristics					
Age, years	32 (26-46)	41 (33-49)	55 (47-61)	65 (56-72)	<0.001
Male sex, n (%)	44 (61)	3 (60)	57 (66)	70 (74)	0.35
Diabetes, n (%)	3 (4)	0 (0)	14 (16)	30 (32)	<0.001
CVD, n (%)	4 (6)	1 (20)	19 (22)	29 (31)	0.001
Smoker, n (%)	2 (3)	0 (0)	7 (8)	13 (14)	0.08
Systolic BP, mmHg	137 (127-151)	147 (147-152)	144 (131-157)	144 (132-164)	0.10
Diastolic BP, mmHg	85 (76-95)	88 (86-93)	85 (75-92)	80 (74-90)	0.21
FRS, %	3.3 (1.3-6.8)	3.4 (1.5-12.4)	14.2 (7.7-24.6)	27.8 (15.9-41.4)	<0.001
Nutritional status					
Malnutrition (SGA>1)	28 (39%)	1 (20%)	25 (29%)	34 (36%)	0.49
BMI, kg/m^2	23.8 (21.5-26.1)	22.2 (19.7-23.5)	24.8 (22.9-27.3)	25.4 (23.5-29.1)	0.002
HGS, % of normal	100 (78-109)	73 (63-82)	89 (71-105)	74 (59-86)	<0.001
Biochemical markers					
Hemoglobin, g/L	110 (101-121)	117 (101-121)	114 (109-121)	113 (104-121)	0.17
Albumin, g/L	35 (32-38)	28 (26-36)	35 (32-38)	32 (28-35)	<0.001
HDL, mmol/L	1.3 (1.1-1.6)	1.7 (1.7-1.9)	1.3 (1.0-1.6)	1.2 (1.0-1.5)	0.016
Triglyceride, mmol/L	1.3 (1.1-2.2)	1.6 (1.6-1.9)	1.5 (1.0-2.0)	1.6 (1.2-2.2)	0.11

Total cholesterol, mmol/L	4.6 (3.8-5.2)	4.8 (3.7-5.0)	4.7 (3.9-5.4)	4.5 (3.6-5.2)	0.86
Calcium, mmol/L	2.3 (2.2-2.4)	2.5 (2.3-2.5)	2.3 (2.2-2.4)	2.3 (2.1-2.4)	0.42
Phosphate, mmol/L	1.6 (1.4-2.0)	2.0 (1.8-2.5)	1.7 (1.5-2.1)	1.8 (1.4-2.1)	0.19
iPTH, ng/L	231 (160-429)	465 (373-522)	274 (179-430)	281 (179-448)	0.42
Inflammatory markers					
hsCRP, mg/L	0.8 (0.3-2.2)	6.5 (1.8-13.9)	1.5 (0.7-3.7)	2.8 (1.0-7.6)	<0.001
IL-6, pg/mL	1.9 (0.5-3.1)	2.3 (1.4-4.5)	2.1 (0.9-6.3)	5.8 (3.3-9.1)	<0.001
AVC and CAC					
AVC score, AU	0	19 (18-22)	0	103 (23-244)	<0.001
CAC score, AU	0	0	134 (14-752)	907 (370-2156)	<0.001
Others					
AGEs, AU	2.8 (2.4-3.3)	3.4 (3.0-3.5)	3.2 (2.9-3.8)	3.5 (2.9-3.9)	<0.001
Aix, %	19.5 (10.9-28.4)	22.0 (1.0-28.5)	22.0 (16.3-28.0)	26.6 (20.3-32.0)	0.004
Medications					
Ca-Blocker, n (%)	36 (50)	3 (60)	40 (46)	52 (55)	0.66
Beta-Blocker, n (%)	33 (46)	3 (60)	57 (66)	76 (80)	<0.001
ACEi/ARB, n (%)	46 (64)	4 (80)	59 (68)	58 (61)	0.69
Statin, n (%)	15 (21)	2 (40)	31 (36)	47 (49)	0.002

Data are presented as median (IQR, interquartile range) for continuous measures, and n (%) for categorical measures; Abbreviations: AVC, aortic valve calcium; CAC, coronary artery calcium; CVD, cardiovascular disease; BP, blood pressure; FRS, Framingham CVD risk score; SGA, subjective global assessment; BMI, body mass index; %HGS, hand grip strength, converted to % of sex-matched healthy controls; HDL, high-density lipoprotein; LDL, low-density lipoprotein; iPTH, intact parathyroid hormone; hsCRP, high sensitivity C-reactive protein; IL-6, interleukin-6; AU, Agatston units; AGEs, advanced glycation end products; AIx(%), augmentation index; ACEi/ARB, angiotensin-converting enzyme inhibitor/ angiotensin II receptor blockers

Supplementary Table 3. Univariate correlations between aortic valve calcium score and other variables (only significant correlations are listed).

	AVC>0	
**Variables**	**Rho**	**p value**
Age	0.52	<.0001
Diabetes	0.24	<.0001
CVD	0.19	0.003
Smoker	0.13	0.04
FRS	0.52	<.0001
Beta Blocker	0.23	0.0002
Statin use	0.20	0.001
%HGS	-0.34	<.0001
Albumin	-0.28	<.0001
Triglyceride	0.15	0.02
hsCRP	0.29	<.0001
IL-6	0.43	<.0001
AGEs	0.25	0.0004
Aortic AIx	0.23	0.0007
CAC score	0.59	<.0001

Abbreviations: AVC, aortic valve calcium; CVD, cardiovascular disease; FRS, Framingham CVD risk score; PEW, protein-energy wasting; %HGS, hand grip strength, converted to % of sex-matched healthy controls; hsCRP, high sensitivity C-reactive protein; IL-6, interleukin-6; AGEs, advanced glycation end products; AIx(%), augmentation index; CAC, coronary artery calcium

Supplementary Table 4. Multivariate logistic regression of factors associated with presence of aortic calcium score (pseudo-R =0.29)

Aortic>0	OR (95% CI)	p-value
per 1-SD increase of FRS	2.25 (1.43, 3.35)	<0.0001
per 1-SD increase of %HGS	0.82 (0.55, 1.21)	0.31
per 1-SD increase of Albumin	0.72 (0.51, 1.04)	0.08
per 1-SD increase of Triglyceride	0.96 (0.69, 1.33)	0.81
per 1-SD increase of hsCRP	1.10 (0.80, 1.50)	0.56
per 1-SD increase of Aix%	0.85 (0.57, 1.26)	0.42
per 1-SD increase of AGEs	1.14 (0.78, 1.67)	0.50
per 1-SD increase of CAC	2.18 (1.34, 3.59)	0.002
Presence of CVD	0.89 (0.39, 2.03)	0.78
Statin use	1.54 (0.80, 2.99)	0.20

Abbreviations: AVC, aortic valve calcium; CVD, cardiovascular disease; FRS, Framingham CVD risk score; %HGS, hand grip strength, converted to % of sex-matched healthy controls; hsCRP, high sensitivity C-reactive protein; CAC, coronary artery calcium; Aix (%), augmentation index; AGEs, advanced glycation end products; CVD, cardiovascular disease

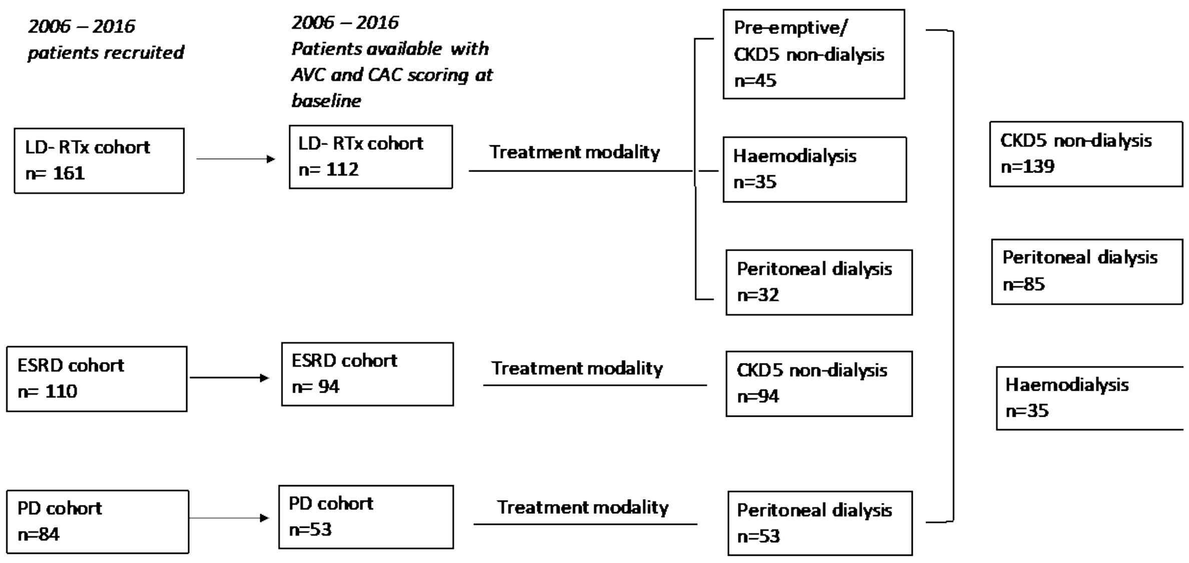

Supplementary Figure 1. Flow chart of patient's recruitment in the cohort study. LD-RTx, living donor kidney transplant

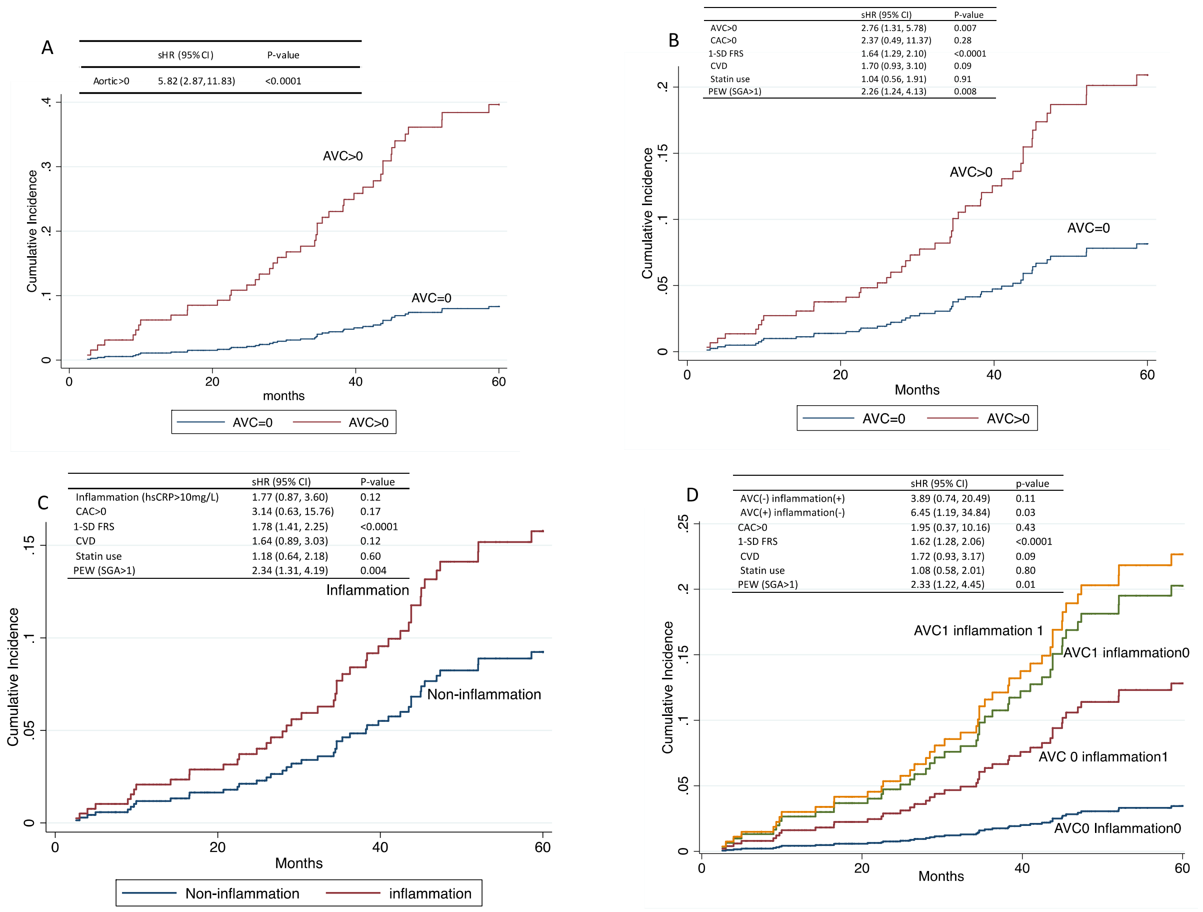

Supplementary Figure 2. Association of aortic valve calcium (AVC) and coronary artery calcium (CAC) with all-cause mortality in separate and combined models. **2A**: AVC and all-cause mortality in crude model **2B**: AVC and all-cause mortality in adjusted model; **2C**: CAC and all-cause mortality in adjusted model; **2D**: Stratification of presence of AVC and CAC with all-cause mortality (AVC (+) CAC (+) group was combined with AVC (+) CAC (-) group due to small size (n=5))

Inserted are multivariate competing risk models for AVC (**B**) CAC (**C**), and stratification AVC and CAC (**D**); all models were adjusted with 1-SD FRS, CVD, inflammation, use of statins and nutritional status (SGA>1).

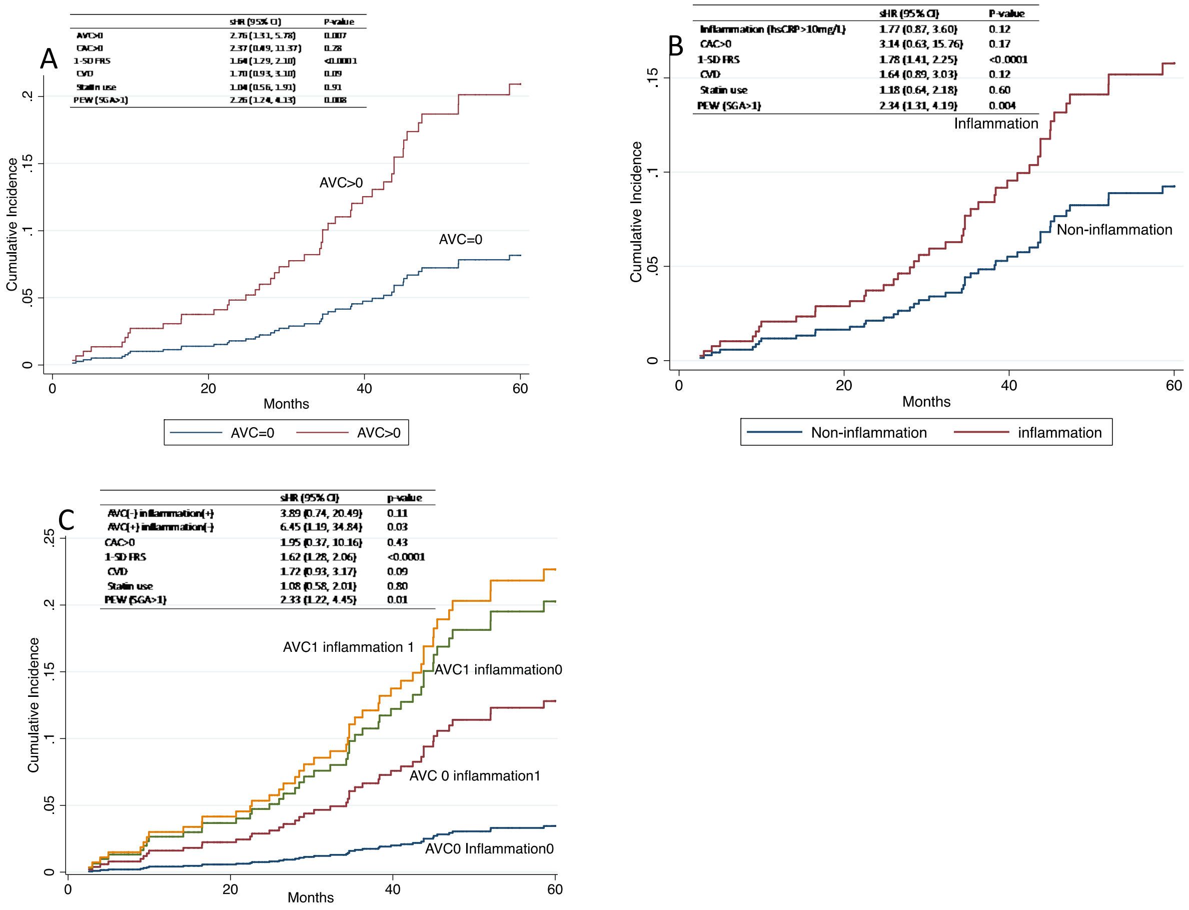

Supplementary Figure 3. Association of aortic valve calcium (AVC) and inflammation with all-cause mortality in separate and combined models. 3A: Presence of AVC and all-cause mortality; 3B: Presence of inflammation and all-cause mortality; 3C: Stratification of presence of AVC and inflammation with all-cause mortality

Inserted are multivariate competing risk models for AVC (A), inflammation (B), and stratification of AVC and inflammation (C); all models were adjusted with 1-SD FRS, CVD, CAC, use of statins and nutritional status (SGA>1).

Supplementary Text 1.

Histological assessment of arterial media calcification

In a subgroup of 102 ESRD patients undergoing LD-Rtx, vascular biopsies were obtained from the inferior epigastric artery[14]. Within 20 minutes after skin incision at the start of surgery, one piece (1-2 cm in length) of the artery was collected by sharp dissection. Samples were immediately placed in All Protect Tissue Reagent (Qiagen, Hilden, Germany), snap-frozen in isopentane and subsequently stored at -70°C, or fixed in 4% phosphate buffered formalin. Formalin-fixed material from the epigastric arteries were embedded in paraffin. 1-2 µm thick sections were stained with hematoxylin and eosin and von Kossa staining, respectively. An experienced pathologist (MS) evaluated the sections. The degree of medial calcification was semi-quantified as no (score 0; n=17), mild (score 1; n=48), moderate (score 2; n=24) or extensive (score 3; n=13) signs of media vascular calcification.

Biochemical assessments

Analyses of plasma hsCRP (coefficient of variation, CV, 5%), calcium, phosphate, intact parathyroid hormone, cholesterol, triglycerides, HDL, cholesterol, creatinine, albumin (CV 3-4%) and hemoglobin were performed at the Clinical Chemical Laboratory of Karolinska University Hospital, Stockholm, Sweden. Plasma interleukin-6, IL-6 (CV 4%) was analyzed by immunometric assays on an Immulite 1000 Analyzer (Siemens Healthcare Diagnostics, Los Angeles, CA, USA) using commercial kits.

Augmentation index (AIx %) measurement

Assessment of arterial stiffness was performed non-invasively by SphygmoCorVR System (AtCor Medical, Sydney, Australia), using tonometry-based and cuff-based SphygmoCorDevices. The peripheral pulse waveform (PPW) was recorded from the radial artery at the wrist in non-fistula arm using applanation tonometry with a sensor probe. PPW and brachial blood pressure measurements were used to estimate central aortic pressure

waveform calculated by the transfer function. Using the cuff-based SphygmoCor Device, brachial artery compression waveforms were obtained by partially inflating a cuff over the brachial artery between the shoulder and the elbow joint. The brachial waveforms were calibrated using cuff-measured brachial systolic and diastolic blood pressures, and then used to generate central aortic pressure waveforms by transfer function. Augmentation pressure (AP) and AIx was derived from this with the technique of pulse wave analysis. The merging of incident and the reflected wave (the inflection point) were identified on the generated central aortic pressure waveform. AP was defined as the maximum systolic pressure minus pressure at the inflection point. AIx was defined as AP divided by pulse pressure and expressed as a percentage. In addition, because AIx is influenced by heart rate, an index normalized for heart rate of 75 beats per minute (bpm) was used. SphygmoCor adjusts the AIx at an inverse rate of 4.8% for each 10 bpm increment.

SAF measurement

Advanced glycation end-products (AGEs) autofluorescence was measured using an Autofluorescence AGE reader (DiagnOptics Technologies BV, Groningen, The Netherlands). Patients with tattooed and dark skin were not investigated. The AGE reader illuminat**es** a skin surface of ~1 cm^2, guarded against surrounding light, with an excitation light source between 300 and 420 nm. Emission light (fluorescence in the wavelength range between 420 and 600nm) and reflected excitation light (with a wave length between 300 and 420nm) from the skin are measured with a spectrometer. SAF was calculated as the ratio between the emission light and reflected excitation light, multiplied by 100, and expressed in arbitrary units (AU). All measurements were performed at room temperature in a semi-dark environment.
